# Influence of Natural Aging on the Moisture Sorption Behaviour of Wooden Structural Components

**DOI:** 10.3390/molecules28041946

**Published:** 2023-02-17

**Authors:** Liuyang Han, Guanglan Xi, Wei Dai, Qun Zhou, Suqin Sun, Xiangna Han, Hong Guo

**Affiliations:** 1 Institute of Cultural Heritage and History of Science & Technology, University of Science and Technology Beijing, Beijing 100083, China; 2 National Center for Archaeology, Beijing 100013, China; 3 Shanxi Provincial Institute for the Protection of Ancient Buildings, Painted Sculptures and Murals, Taiyuan 030000, China; 4 Department of Chemistry, Tsinghua University, Beijing 100084, China

**Keywords:** historical wooden buildings, water vapor sorption, aged wood, FT-IR spectroscopy, two-dimensional correlation infrared spectroscopy

## Abstract

A greater understanding of moisture sorption behaviour of aged wooden structural components, which has a close relationship with dimensional stability, is required to effectively evaluate and preserve historical artefacts. This study focused on the effects of aging on Baotou beam samples from a Chinese historical wooden building. An analysis of the sorption isotherms and hysteresis loops of a naturally aged, decayed sample (AOS), an aged sound sample (AIS), and a reference sample (RS), using classical sorption isotherm models revealed that the moisture sorption behaviour of samples from the same growth ring in a Baotou beam can differ significantly. AOS showed higher hygroscopicity than AIS, and both these samples were more hygroscopic than RS. Furthermore, the mono/multilayer moisture contents of AOS were always higher than those of AIS and RS. In addition, Fourier transform infrared, second-derivative infrared, and two-dimensional correlation infrared spectroscopy were used to investigate chemical changes in the samples. The relative hemicellulose and lignin contents of the samples changed significantly with wood aging. Furthermore, AOS exhibited the highest calcium oxalate content, which may be associated with fungal infections. Overall, these results provide valuable insights into the effects of aging on wood samples and the dimensional stability of timber structures, which could inform future research on methods for the preservation or restoration of aging timber structures.

## 1. Introduction

The protection of ancient wooden buildings in China and overseas is of great importance due to their cultural and historical significance [1,2]. Such structures provide invaluable insights into the science, technology, and living conditions of past eras, making them an irreplaceable part of our cultural heritage [3]. As these buildings are vulnerable to environmental factors, which cause weathering and aging, specific preservation and conservation efforts are essential to ensure their survival for future generations [1,4]. Accordingly, the protection of ancient wooden buildings has received worldwide attention, with various initiatives and projects implemented to preserve these important structures [5,6,7], including the development of detailed research and conservation plans [8,9], the implementation of protective measures such as waterproof coatings and surface treatments [10,11], and careful monitoring of these buildings to identify any signs of deterioration [12,13]. Additionally, traditional construction techniques and materials have been studied to gain insights into optimal restoration and conservation methods for ancient structures [5,14].

In recent years, studies on ancient timber structures and archaeological artefacts have focused on material durability, structural design, and physical performance [15,16]. In particular, the influence of water and moisture on the safety and stability of archaeological artefacts has been investigated [11]. Archaeological wood has been shown to be particularly vulnerable to degradation at ambient temperature and humidity levels because of its biodegradability and hygroscopicity [1,4,17]. In addition, degradation of the main components of wood, such as lignin and cellulose, can further increase hygroscopicity, leading to decreased dimensional stability and reduced protection against decay. To manage this issue, an understanding of the influence of natural aging on moisture sorption behaviour is essential [4]. Studies have indicated that the deterioration process can significantly change the hygroscopicity of wood, with differences observed depending on the species and sample size [18,19]. These changes also affect other physical properties, such as shrinkage and swelling, making it difficult to ensure the optimum performance and durability of aged wood components [20]. In addition, research on the protection and conservation of ancient timber structures has shown that appropriate monitoring and testing methods are critical for maintaining the structural integrity of aged wood components [12,21]. Despite the importance of the relationship between natural aging and the moisture sorption behaviour of wood materials for developing effective solutions to protect cultural relics [1,4,11,17], few studies have been conducted in this area.

To address the above-mentioned issues, suitable methods for assessing the degradation of wooden artefacts are required. For the sorption behaviour of wooden beams, dynamic vapour sorption (DVS) can be applied to evaluate the equilibrium moisture content (EMC) and hygroscopic characteristics [22,23,24]. However, most DVS instruments can only test a single sample per experiment, which can introduce testing errors during multiple measurements of different samples. Recently, the relationship between waterlogged archaeological wood and hygroscopicity has been investigated using simultaneous DVS. With this technique, up to 23 wood samples can be evaluated simultaneously under the same environmental conditions, thus providing increased accuracy and facilitating comparisons [25]. Furthermore, various chemical techniques are available for assessing the degradation of wooden artefacts, including wet chemistry [1,26], infrared (IR) spectroscopy [27,28,29], near-infrared (NIR) spectroscopy [30,31], Raman spectroscopy [27,32], nuclear magnetic resonance (NMR) spectroscopy [27,33,34], gas chromatography–mass spectrometry (GC-MS) [27,35,36], direct analysis in real time mass spectrometry (DART-MS) [37,38], and X-ray techniques [27,39,40]. IR spectroscopy, which is among the most popular methods for determining the chemical structures of wood, can provide information about the disappearance or generation of various functional groups or chemical bonds based on peak position, shape, and intensity [28]. This method also allows semi-quantitative analysis of the relative contents of the three main chemical components of wood (cellulose, hemicellulose, and lignin) using the signal intensities of their characteristic peaks [27]. Despite the advantages of IR spectroscopy, the investigation of complex samples, such as archaeological wood, is challenging owing to the superposition of the absorption signals corresponding to different components [41,42]. Two-dimensional correlation infrared (2D COS-IR) spectroscopy, which was proposed by Noda in 1986, has been widely used to analyse environmental samples, natural products, polymers, foods, proteins and peptides, solutions, mixtures, and complex traditional Chinese medicines [43,44]. In the field of wood, this technique has mainly been applied to wood identification and to evaluate biodegraded wood [41,44,45,46]. However, it has not been used to study archaeological wood from historical wooden buildings. 

This study investigated the effects of natural aging on the hygroscopic properties of wood in historical wooden buildings. The Baotou beam, which is a typical load-bearing structural component in ancient buildings, was used as the research object. Sorption isotherms and hysteresis loops obtained using DVS were analysed using the classical Guggenheim-Anderson-de Boer (GAB) and Hailwood-Horribon (H-H) models to characterize the sorption behaviour of naturally aged samples. In addition, chemical changes in the samples were investigated using Fourier transform infrared (FT-IR), second-derivative infrared (SD-IR), and 2D COS-IR spectroscopy.

## 2. Results and Discussion

### 2.1. Morphology of the Aged Wooden Beam

**Figure 1:**
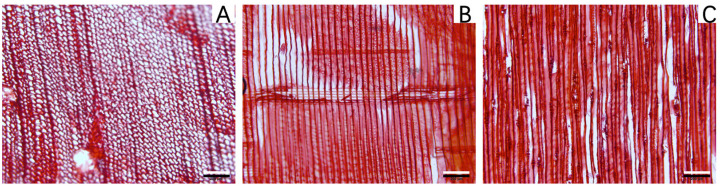
Morphological structure of the wooden Baotou beam: (**A**) cross-section, (**B**) radial section, and (**C**) tangential section. Scale bars = 200 μm.

For this study, a Baotou beam from Sanshen Temple (Changzhi City, Shanxi Province, China) was selected as an aged wooden structural component. Two samples were taken from the same growth ring of the Baotou beam, one from the outermost layer (archaeological outer-location sample, AOS) and the other from a location with a layer of growth rings on the outer surface (archaeological inner-location sample, AIS). The morphology of a sample from the wooden beam is shown in Figure 1. Various anatomical features (International Association of Wood Anatomists (IAWA) indices: 4, 26, 33, 40, 43, 44, 79, 82, 85, 89, 90, 98, 104, 107 and 109) were observed [47]. These were the most prominent characteristics for wood identification and included growth rings (IAWA index 40), an earlywood to latewood gradient (IAWA index 43), cross-sections of earlywood tracheids consisting of irregular squares, rectangles, and polygons, cross-sections of latewood tracheids consisting of rectangles and squares (Figure 1A), tracheids bordered by a single row of round pits in the radial walls (IAWA index 44), window-like cross-field pitting, end walls of ray parenchyma cells smooth, indentures present (Figure 1B)(IAWA indices 79, 82, 85, 89 and 90), number of pits per cross-field 1–3; (IAWA index 98), tracheids bordered by obvious pitting in the tangential walls and uniserial and spindle rays with heights of 16–30 cells, and axial intercellular (resin) canals present (Figure 1C)(IAWA indices 104, 107 and 109). Based on these microscopic characteristics, the wood block from the wooden beam was identified as *Pinus* sp. (pine wood) [47]. For comparison with the aged wooden beam, a recent *Pinus* sp. specimen (Longyan City, Fujian Province, China) was selected as a reference sample (RS).

### 2.2. Moisture Sorption Behaviour

**Figure 2:**
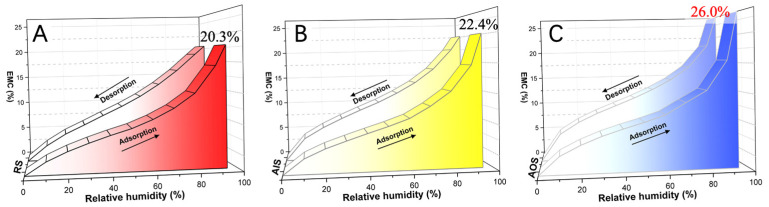
Multistep sorption isotherms of (**A**) the reference sample (RS), (**B**) the archaeological inner-location sample (AIS), and (**C**) the archaeological outer-location sample (AOS).

The multistep sorption isotherms of AIS, AOS, and RS are shown in Figure 2. Although AIS and AOS were obtained from the same annual ring, AOS was located on the outermost part of the wooden beam, whereas some growth rings were located on the outer surface of AIS. Based on the sorption mechanism, the measured isotherms may be type IV or type II, corresponding to mostly monolayer or multilayer adsorption or to a mixture of these processes with capillary condensation in the mesopores of the cell wall, respectively [24]. All the adsorption and desorption isotherms were S-shaped, which is characteristic of International Union of Pure and Applied Chemistry (IUPAC) type II isotherms [48,49]. In Figure 2, all the samples showed an upward slope from approximately 50% relative humidity (RH), which is consistent with previous studies on lignocellulosic materials [24,50]. In addition, the EMC values of the archaeological samples (AOS and AIS) at each RH during the adsorption and desorption stages were greater than those of RS. The maximum EMC values (at 95% RH) of AIS and AOS were 22.4% and 26.0%, respectively, whereas that of RS was only 20.3%. Thus, the EMC values of AIS and AOS were 10.3% and 28.1% higher, respectively, than that of RS. Although AOS and AIS were both obtained from near the same growth ring in the wooden beam, the EMC values of AOS were almost 16.1% higher than those of AIS at each RH.

**Figure 3:**
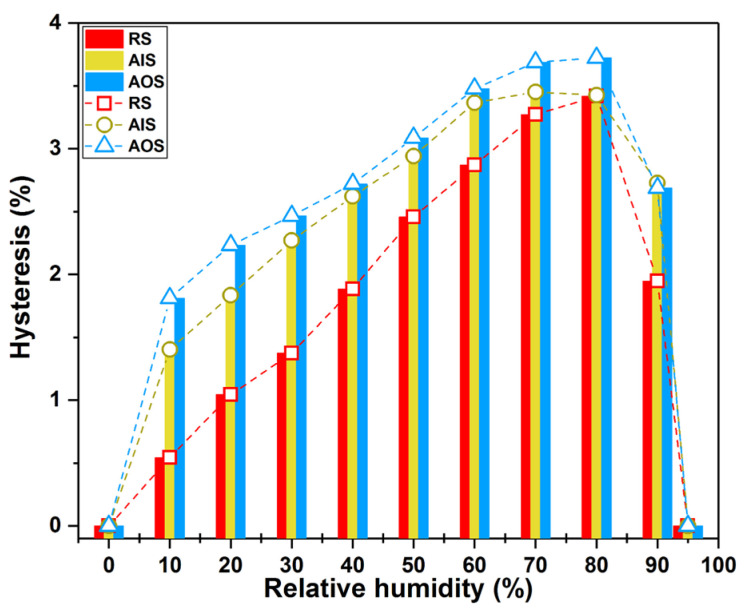
Sorption hysteresis of RS (red), AIS (yellow), and AOS (blue).

In addition, AIS and AOS exhibited different sorption hysteresis. As described previously [42,51], the sorption hysteresis was calculated as the difference between the adsorption and desorption branches of the isotherm at each RH [52]. In this study, the sorption hysteresis of AOS, AIS, and RS was obtained for a moisture range from 0% RH to 95% RH. As shown in Figure 3, the hysteresis of AOS and AIS was higher than that of RS. Furthermore, AOS had higher hysteresis than AIS at each RH, which indicates that the outer part of the wooden beam had a greater degree of decay than the inner part. The surface moisture content may vary more in naturally aged AOS than in relatively sound AIS because the sorption hysteresis is greater under the same RH conditions [24,53], which could detrimentally affect the dimensional stability of wooden beams.

To further clarify the sorption behaviour of the naturally aged samples, the adsorption and desorption isotherms were fitted to the classical GAB and H-H models. The fits were considered valid when all the determination coefficient (*R*^2^) values were greater than 0.99 [24,54,55,56]. The parameters calculated by least-squares fitting are listed in Table 1.

The *R*^2^ values for the GAB model were all greater than 0.99 (Table 1), indicating that this model could correctly describe the relationship between RH and EMC for AIS, AOS, and RS during the sorption process [24,42,55]. It should be noted that the conjunction of the relations 5.57 ≤ *C*_GAB_ < ∞ and 0.24 < *K*_GAB_ ≤ 1 was satisfied for the sorption isotherms of all the samples, confirming their classification as IUPAC type II [24,49,57]. As shown in Table 1, the *C*_GAB_ values were approximately 10 times greater than the *K*_GAB_ values, indicating that monolayer sorption required much more heat than multilayer sorption [24,58]. In particular, the *C*_GAB_ coefficients, which represent the total heat of sorption for monolayer water [54,59], were higher for the archaeological wood samples than for RS. The *C*_GAB_ coefficients of AOS were 75.60% and 84.94% higher than those of AIS during adsorption and desorption, respectively. Furthermore, the *C*_GAB_ coefficients of AIS were 51.91% and 141.20% higher than those of the RS during adsorption and desorption, respectively. These results indicate that the monolayer water in AOS had a higher binding energy with multilayer water molecules [60] than that in AIS. 

**Figure 4:**
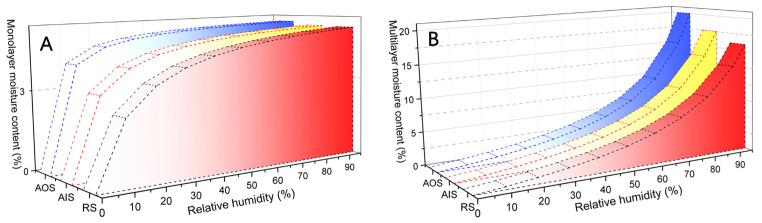
Moisture content at each relative humidity of the H-H model. Histogram for monolayer moisture content (**A**) and multilayer moisture content (**B**) at each RH.

The *R*^2^ values for the H-H model were all greater than 0.99 (Table 1), confirming the validity of the fitting results [24,42,55]. The molecular weight of wood at every adsorption site (*w*) reflects the extent of monolayer water sorption at each RH. AOS had the highest *w* value (Table 1), indicating that the decayed archaeological sample could hold more monolayer water at each RH. Thus, the *M*_h_ values, which reflect the monolayer moisture content, of AOS and AIS were always greater than those of RS (Figure 4A) over the entire RH range. Nonetheless, the monolayer moisture contents of AIS and RS were similar, indicating that the capacities of AIS and RS were also similar (Figure 4A and Table 1). For the archaeological samples, the monolayer moisture content of AOS was greater than that of AIS at each RH, indicating that relatively decayed AOS always absorbed more monolayer water than relatively sound AIS. The monolayer water content dramatically increased at relatively low humidities (0–30% RH) and then stabilised at 40% RH (Figure 4A), with *M*_h_ values of 3.54–4.15%, 3.76–4.17%, and 4.09–4.29% for RS, AIS, and AOS, respectively, at 40–95% RH. The *M*_s_ values, which reflect the multilayer moisture content, increased with increasing RH, and the rate of increase also increased from 0% to 95% RH (Figure 4B). At less than 40% RH, the *M*_h_ values of RS, AIS, and AOS were less than 1.52%, 1.52%, and 1.55%, respectively. At 40–80% RH, which corresponds to the annual average monthly variation in humidity at the location of the wooden building from which the archaeological samples were obtained, the *M*_h_ values of RS were 2.28–8.79%, whereas those of AIS and AOS were 2.28–9.16% and 2.34–9.86%, respectively. Above 80% RH, the *M*_h_ values of RS were 8.79–16.01%, whereas those of AIS and AOS were 9.16–17.55% and 9.86–20.05%, respectively (Table 1 and Figure 4B). Thus, if the environmental humidity is higher than the humidity of the wooden structure, the multilayer water content may increase exponentially. At 95% RH, AIS and AOS absorbed 9.5% and 25.2%, respectively, more multilayer water than RS.

The analysis of samples from regions near the same growth ring in the wooden beam using the GAB and H-H models revealed that the hygroscopicity of the relatively aged sample (AOS) was higher than that of the relatively sound sample (AIS). Furthermore, the EMC value of AIS was lower than that of AOS at each RH, and this difference became more pronounced when the RH exceeded the upper limit of the wooden beam for storing moisture (80% RH) (Figure 2 and Figure 4). In wooden structures, such as wooden beams, to achieve an EMC, water vapour moves from high-humidity to low-humidity regions [42,61], that is, from highly decayed regions to relatively sound regions. Under the same humidity conditions, the EMC and amount of adsorbed water for AOS were greater than those for AIS, which were greater than those for RS. Although AOS and AIS were obtained from the same growth ring in the wooden beam, they contained different amounts of adsorbed water, especially multilayer adsorbed water vapour, at each RH. As the contents of both monolayer and multilayer moisture in the cell walls of wood are mainly related to its cell wall structure [55], AOS with high mono/multilayer moisture contents may undergo greater dimensional changes than AIS upon humidity change.

The archaeological samples exhibited differences in hygroscopicity, despite being selected from regions near the same growth ring in the wooden beam. The GAB and H-H sorption models both indicated that the sample selected from the outer position possessed more sorption sites and had a greater capacity for adsorbing water vapour from the surrounding environment than the sample selected from the inner part. Previous research has suggested that aging may cause changes in the chemical compositions of wooden structural components [4]; thus, to clarify the observed differences between AOS and AIS, the samples were systematically investigated using various IR spectroscopic techniques.

### 2.3. Chemical Changes

**Figure 5:**
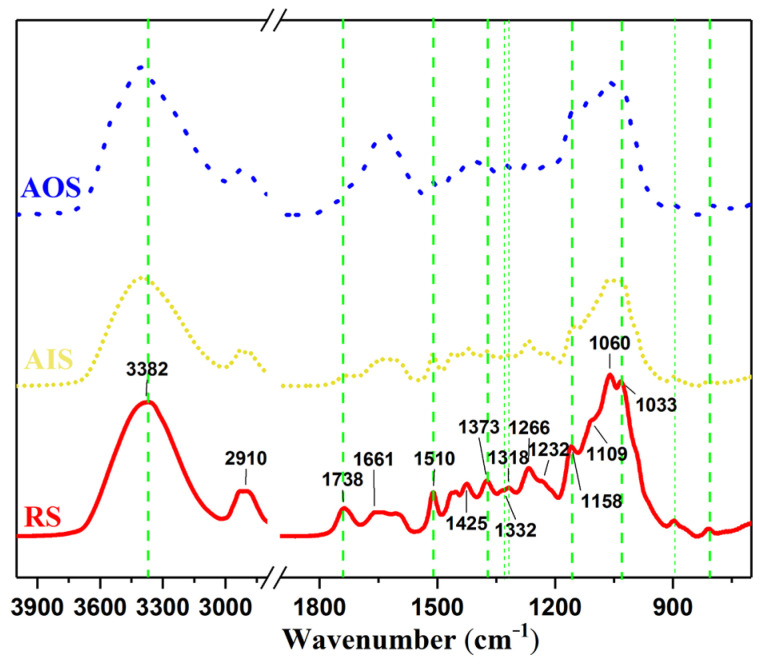
FT-IR spectra of RS (red), AIS (yellow), and AOS (blue).

**Figure 6:**
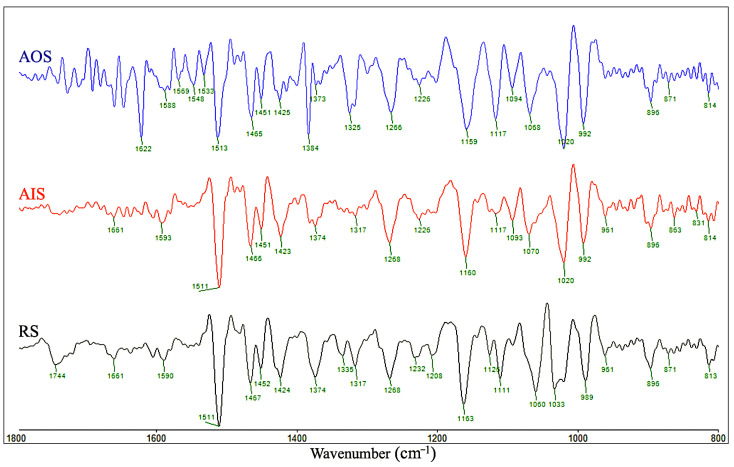
SD-IR spectra of RS (black), AIS (red), and AOS (blue).

FT-IR and SD-IR spectroscopy were used to reveal the chemical changes in the archaeological samples from different positions near the same growth ring in the wooden beam. The FT-IR and SD-IR spectra of AOS, AIS, and RS are shown in Figure 5 and Figure 6. The assignments of the main peaks in the FT-IR spectra are summarized in Table 2. Overall, the relative contents of hemicellulose and lignin in the samples changed significantly with increasing wood aging. Specifically, the peak at 1737 cm^−1^, which is ascribed to the C=O stretching vibrations of acetyl and carbonyl groups in hemicellulose, decreased significantly in intensity from RS to AIS and AOS, indicating that the hemicellulose content decreased with aging. Moreover, the peak corresponding to CH deformation and CH_3_ symmetric deformation in polysaccharides shifted from 1373 cm^−1^ for AIS to 1384 cm^−1^ for AOS.

Although the FT-IR spectra of AOS and AIS appeared similar, their correlation coefficients for comparison with RS were significantly different (0.9248 and 0.6214, respectively; Table 3). This difference may be due to the complexity of the fingerprint region (1800–800 cm^−1^), where different wood components contribute to many overlapping bands [42]. Thus, the significantly different coefficients of AOS and AIS indicate that these samples exhibit differing degrees of degradation.

Two-dimensional COS-IR spectroscopy is a spectral analysis technique based on cross-correlation calculations [64]. A perturbation (such as temperature) is applied to the sample to obtain a series of dynamic spectra. Subsequently, cross-correlation calculations are performed to produce a two-dimensional correlation spectrum. As different absorption peaks have different sensitivities to perturbation, 2D COS-IR spectra provide additional information that cannot be obtained from the corresponding FT-IR spectra [65]. In particular, in 2D COS-IR spectra, the apparent resolution is improved and information about interactions between functional groups in the molecules of the sample and related intermolecular interaction can be obtained [42,65]. To further elucidate the effect of aging on wooden beams, 2D COS-IR spectra of AOS, AIS, and RS were collected, and the contour maps in the regions of 900–1560 cm^−1^ and 1560–1800 cm^−1^ are shown in Figure 6. In the 2D COS-IR spectral plots, regions that undergo a greater change in intensity show stronger autopeaks than those that remain constant [42,64,66]. As different chemical components in the wood samples may react differently to temperature perturbation, the differences in the position and intensity of the autopeaks for AOS, AIS, and RS reflect the degree of aging.

**Figure 7:**
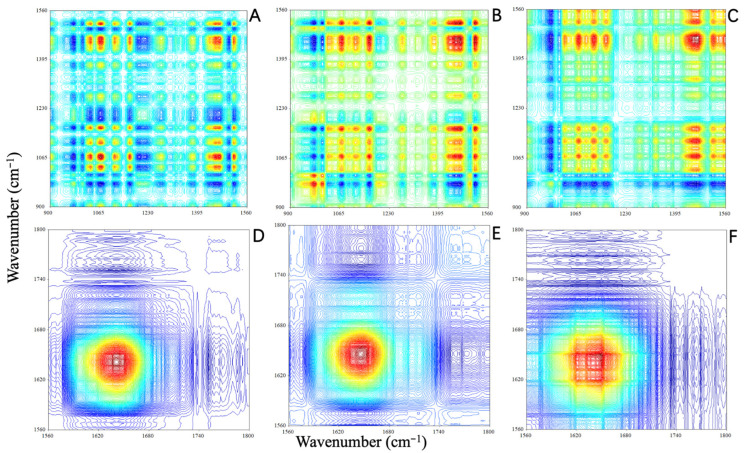
Synchronous 2D COS-IR images of RS, AIS, and AOS in the range of (**A**–**C**) 900–1560 cm^−1^ and (**D**–**F**) 1560–1800 cm^−1^ (red: positive; blue: negative).

In the region of 900–1560 cm^−1^, the 2D COS-IR spectra of the archaeological samples (AIS and AOS) and RS showed distinct differences (Figure 7A–C). In detail, RS exhibited 14 obvious autopeaks in the synchronous 2D COS-IR image in the range of 900–1560 cm^−1^ (982, 1034, 1070, 1115, 1165, 1195, 1231, 1276, 1379, 1434, 1453, 1467, 1500, and 1515 cm^−1^; Figure 7A). The positions of these peaks were consistent with the SD-IR spectra (Figure 6). The autopeaks related to cellulose and hemicellulose at 1034, 1070, 1115, 1165, 1379, 1434, 1453, and 1467 cm^−1^ were strong, indicating that the functional groups in cellulose and hemicellulose are highly sensitive to temperature perturbation [41,42,67]. Furthermore, the strong autopeak at 1515 cm^−1^ (Figure 7A) was attributed to the stretching vibration of the heterocyclic skeleton in lignin. The cross-peaks between the aforementioned autopeaks were positive, that is, the intensities of these peaks increase or decrease with increasing temperature [41,65]. In contrast, several autopeaks related to lignin (982, 1195, 1276, and 1500 cm^−1^) were weaker (Figure 7A), indicating that the corresponding functional groups did not change significantly with temperature [42,67]. The cross-peaks between these peaks and those of cellulose and hemicellulose at 1034, 1070, 1115, 1165, 1379, 1434, 1453, and 1467 cm^−1^ were negative, indicating that the guaiac ring and C–O stretching vibrations in lignin were less sensitive to temperature perturbation [64,68]. AIS showed 11 obvious autopeaks in the range of 900–1560 cm^−1^ (Figure 7B) located at 982, 1011, 1066, 1115, 1163, 1270, 1382, 1448, 1465, 1499, and 1513 cm^−1^. All the peaks were positively correlated, except for the cross-peaks correlated with 982, 1011, and 1499 cm^−1^. For AOS, 10 autopeaks were observed at 980, 1024, 1066, 1116, 1165, 1273, 1329, 1382, 1465, and 1515 cm^−1^ (Figure 7C). With the exception of the peak at 980 cm^−1^, which was attributed to the C–O and C–C stretching of >CH-OH and -CH_2_OH groups in cellulose [45,69], the autopeaks exhibited positive cross-peaks, indicating increased intensities with increases in temperature.

In the region of 1560–1800 cm^−1^, RS and AIS exhibited a single autopeak at 1640 cm^−1^, corresponding to the deformation vibration of water. For AOS, an additional peak was observed at 1620 cm^−1^, as also reflected in the corresponding SD-IR spectrum (Figure 6). In combination with the observation of absorption peaks at 1323 and 781 cm^−1^ in the FT-IR spectrum, this result suggests that there was a small amount of calcium oxalate in AOS. Calcium oxalate has previously been observed in wood matrices in an advanced stage of decay [70], which confirms that the degree of decay for AOS was more severe than that for AIS. Moreover, calcium oxalate in wooden components from historical buildings may be related to fungal activity [71,72]. As AOS was exposed to the outermost part of the wooden component, a stronger calcium oxalate signal was observed for this sample than for AIS, likely because fungal infections of wood components often proceed from external to internal regions [73,74,75]. 

Overall, naturally aged wood becomes increasingly sensitive to thermal perturbation as the degree of degradation increases. AOS was more sensitive to thermal perturbation than AIS, which may have been caused by the degradation of hemicellulose side chains, resulting in the degree of decay for AOS being more severe than that for AIS, as suggested by the disappearance of the peak at 1738 cm^−1^ in AOS. Wood deterioration under moist conditions is usually accompanied by softening of the woody tissue surface due to the specific action on carboxyl and acetyl groups in hemicelluloses [45]. We confirmed that the natural aging of the wooden structural component proceeded with the deterioration of hemicellulose, as indicated by the disappearance of acetyl and carbonyl groups in AOS [4,23,42]. The differences in the sorption behaviour of samples from regions near the same growth ring in the wooden beam might be related to these chemical changes. The chemical deterioration of the outermost part of the wooden beam resulted in the greatest increase in EMC, hysteresis, and mono/multilayer moisture content at each RH, which may influence the dimensional stability of wooden components [4,42,76].

## 3. Materials and Methods

### 3.1. Materials

**Figure 8:**
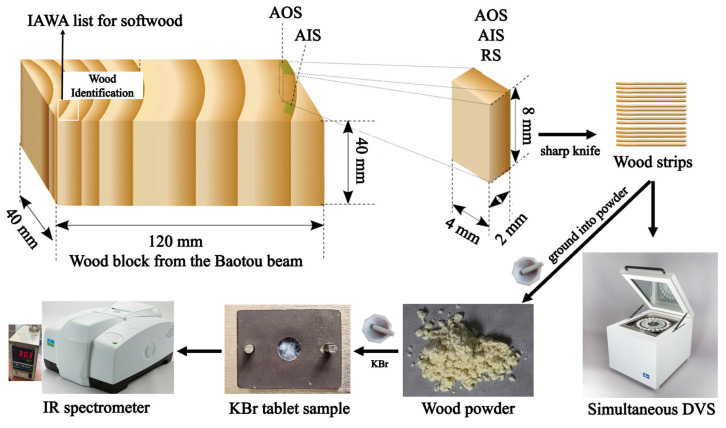
Schematic of sample preparation and measurements.

Sanshen Temple, a historical building located in Changzhi City, Shanxi Province, China (36°11′ N and 113°59′ E), has a Yuan Dynasty style and was built in using ancient Ming Dynasty (1368–1644 A.D.) architecture. Many decayed wooden structural components in the ancient temple have been repaired, and a wooden Baotou beam was replaced during a recent renovation in 2019. A wooden block with a thickness and width of 4 cm and a length of approximately 12 cm was collected from the visually naturally aged part of the Baotou beam. Two samples were carefully selected from the same growth ring of the Baotou beam: one was taken from the outermost layer of the Baotou beam (archaeological outer-location sample, AOS), and the other had a layer of growth rings on its outer surface (archaeological inner-location sample, AIS) (Figure 8). Based on the wood identification results for the aged wooden beam, a sound wood specimen (*Pinus* sp. from Longyan City, Fujian Province, China (25° N, 115° E), 390 m a.s.l., 35 years old, 30 cm in diameter at breast height) was selected as a reference sample (RS).

### 3.2. Methods

#### 3.2.1. Wood Identification

For wood identification, wood samples with a side length of 1 cm were cut from the Baotou beam (Figure 8). The general wood identification process has been described previously [27,42]. Briefly, the samples were cut (15–20 μm in thickness) using a rotary slicer (Leica Autocut, Leica, Germany). Then, optical microscopic slices were prepared by dyeing, dehydration, transparency, and sealing [27]. The anatomical structure was observed using an optical microscope (BX 50; Olympus, Japan). Finally, the anatomical structure was compared with the IAWA list for softwood and then identified by comparison with the wood specimens and slices in the Wood Collection of the Chinese Academy of Forestry (http://bbg.criwi.org.cn (accessed on 20 December 2021)) [27,77].

#### 3.2.2. DVS Measurements

The EMC values of the wood samples at each RH were measured by simultaneous DVS (SPSx-1µ-HighLoad, ProUmid, Germany) using a previously described protocol [24,42]. Briefly, the samples (25 mg) were cut into millimetre-thick strips using a sharp knife (Figure 8). Then, the samples were exposed to increasing RH from 0% to 95% (at intervals of 10% from 0–90% RH) at 25 °C for adsorption measurements. Subsequently, the samples were exposed to decreasing RH in the same manner for desorption measurements. For each step, equilibrium was defined as a mass change per time (dm/dt) of less than 0.0001%/min.

#### 3.2.3. Sorption Isotherm Analysis

The classical GAB and H-H models were fitted to the sorption isotherm data to determine the sorption behaviour of the wood samples. The model parameters were calculated using Origin 2022 software (OriginLab Corporation, Northampton, MA, USA). For the GAB model, the following formula was used [24]:

(1) $$EMC=M_{m}\frac{K_{\mathrm{GAB}}•C_{\mathrm{GAB}}•RH}{\left(1-K_{\mathrm{GAB}}•RH\right)•\left(1-K_{\mathrm{GAB}}•RH+C_{\mathrm{GAB}}•K_{\mathrm{GAB}}•RH\right)}\times 100\%$$

where *EMC* (%) is the equilibrium moisture content, *RH* (%) is the relative humidity, *M*_m_ is the monolayer capacity, *C*_GAB_ (%) is the equilibrium constant related to monolayer sorption, and *K*_GAB_ (%) is the equilibrium constant related to multilayer sorption [24].

For the H-H model, the following equation was used [42]:

(2) $$EMC=M_{h}+M_{s}=\frac{1800}{w}\cdot \frac{k_{1}\cdot k_{2}\cdot RH}{100+k_{1}\cdot k_{2}\cdot RH}+\frac{1800}{w}\cdot \frac{k_{2}\cdot RH}{100-k_{2}\cdot RH}\times 100\%$$

where *EMC* (%) is the equilibrium moisture content, *RH* (%) is the relative humidity, *M*_h_ is the monolayer moisture content (%), *M*_s_ is the multilayer moisture content (%), *w* is the molecular weight of wood at every adsorption site, and *k*_1_ and *k*_2_ are equilibrium constants for the sorption process [55].

#### 3.2.4. FT-IR and SD-IR Spectroscopy

IR spectra of RS, AIS, and AOS were recorded using a PerkinElmer Spectrum IR spectrometer (PerkinElmer, Massachusetts, MA, USA) equipped with a deuterated triglycine sulfate (DTGS) detector. The spectra were collected from 4000 to 400 cm^−1^ with a resolution of 4 cm^−1^ and 16 scans were accumulated [42]. Each sample (approximately 1–2 mg) was blended with KBr (100 mg), ground into powder, and pressed to form a tablet (Figure 8) [42,46]. Interference from H_2_O and CO_2_ was subtracted during scanning. The IR and SD-IR spectra were calculated using the 13-point Savitzky–Golay algorithm in the PerkinElmer Spectrum software (version 10.6.3, PerkinElmer, Massachusetts, MA, USA) [41]. The data were processed using the PE Spectrum software and Origin 2022 software (OriginLab Corporation, Northampton, MA, USA) [42,65]. The correlation coefficients between the archaeological samples (AIS and AOS) and RS were calculated using the PE Spectrum software [42].

#### 3.2.5. 2D COS-IR Spectroscopy

A temperature controller (CKW-1110, Beijing Chaoyang Automation Instrument Co., Beijing, China) was used to obtain a series of dynamic spectra under thermal perturbation. The temperature range was 50–120 °C at a heating rate of 2 °C/min, and IR spectra were collected at intervals of 10 °C (Figure 8) [42]. The 2D COS-IR spectra were produced using 2D correlation analysis software developed by Tsinghua University (Beijing, China) [46,65]. The autopeaks on the diagonal line showed self-correlation and the sensitivity of typical functional group vibrations to thermal perturbation [42]. As the functional groups became more sensitive, the spectra became redder with stronger autopeak intensities in Figure 7 [67].

## 4. Conclusions

This study demonstrated the efficacy of simultaneous DVS and 2D COS-IR techniques for evaluating the degradation of naturally aged wooden beams. The changes in hygroscopic behaviour and chemical properties caused by the natural aging of the wooden beam were investigated. For the selected wooden beam, which was anatomically identified as *Pinus* sp. (pine wood), simultaneous DVS revealed differences in the hygroscopic behaviour of samples from the same growth ring. Moreover, both the archaeological samples were more hygroscopic than a recent wood specimen. Two-dimensional COS-IR spectroscopy analysis indicated that the observed hygroscopic differences originated from differences in the chemical structures of the samples taken from different locations in the Baotou beam. In particular, the increased number of moisture sorption sites in the naturally aged sample taken from the outermost layer could explain the increased hygroscopicity and sorption hysteresis over the entire RH range. 

Although this study provides proven methods and primary knowledge for the evaluation and preservation of historical wooden buildings, further investigations are required to elucidate the relationship between the hygroscopicity and chemical properties of archaeological wood samples.

## Figures and Tables

**Table 1:** Coefficients of the GAB and H-H models for AOS, AIS, and recent wood.

Sample	Sorption Phase	GAB Model				H-H Model				
		*M*_m_	*C*_GAB_	*K*_GAB_	*R* ^2^	*w*	*k*_1_	*k*_2_	*M*_h_	*M*_s_
RS	Adsorption	4.82	9.44	0.80	0.999	379.36	9.08	0.81	4.15	16.01
	Desorption	9.38	5.34	0.62	0.999	195.19	4.45	0.63	6.71	13.81
AIS	Adsorption	4.57	14.34	0.83	0.995	397.80	14.78	0.83	4.16	17.55
	Desorption	7.61	12.88	0.70	1	235.81	11.73	0.70	6.77	15.51
AOS	Adsorption	4.50	25.18	0.85	0.997	404.88	33.32	0.86	4.28	20.05
	Desorption	7.29	23.82	0.74	0.991	251.76	25.91	0.75	5.69	18.05

Note: *M*_m_ is the monolayer capacity, *C*_GAB_ is the equilibrium constant associated with monolayer sorption, *K*_GAB_ is the equilibrium constant related to multilayer sorption [24], *w* is the molecular weight of wood at every adsorption site, *k*_1_ and *k*_2_ are equilibrium constants for the sorption process, *M*_h_ is the monolayer moisture content (%), *M*_s_ is the multilayer moisture content (%) [42,55]. RS: reference sample, AIS: archaeological inner-location sample, AOS: archaeological outer-location sample.

**Table 2:** Peak assignments for the FT-IR spectra of the wood samples in the fingerprint region (1800–800 cm^−1^) [4,42,45,62,63].

Peak Position/cm^−1^			Vibration Mode	Peak Assignment
RS	AIS	AOS		
1738	1736	-	C=O stretching of acetyl and carbonyl groups	hemicelluloses
1661	1661	1661	the relative concentration of aromatic skeletal vibrations, together with C=O stretch	lignin
1639	1626	1634	Deformational mode	H_2_O
-	-	1622	C=O-O stretching	calcium oxalate
1590	1593	1588	C=C stretch of substituted aromatic ring	Lignin (in SD-IR)
1510	1510	1510	C=C stretch of substituted aromatic ring	lignin
1453	1458	-	CH_3_ asymmetric stretch, CH_2_ scissoring	lignin/carbohydrates
1425	1423	1415	CH_2_ scissoring	cellulose
1373	1375	1384	CH deformation, CH_3_ symmetric deformation in	cellulose/hemicelluloses
1318	1318	1323	C-O-H stretch, the wagging (out of the plane) of the CH_2_ groups	calcium oxalate/crystalline cellulose
1266	1268	1267	Aromatic C-O stretching vibrations of methoxyl and phenyl propane units in guaiacol rings	lignin
1232	1226	1226	mainly assigned to the C-O stretching in the O=C-O group of side chains	hemicellulose
1158	1154	1152	Asymmetric bridge C-O-C stretch mode	cellulose/hemicelluloses
1109	1110	1115	C-OH in plane deformation	cellulose/hemicelluloses
1060	1049	1063	C-O stretching mainly from C (3)-O (3)H	cellulose I
1033	1033	1033	C-O and C-C stretch of > CH-OH and –CH_2_OH groups	cellulose
982	982	980	C-O and C-C stretch of > CH-OH and -CH_2_OH groups	Cellulose (in 2D COS-IR)
897	897	897	the β-(1→4)-glycosidic linkage	cellulose

**Table 3:** Correlation coefficients for the comparison of AOS and AIS with RS between 1800 and 800 cm^−1^.

Sample	Correlation Coefficients
RS	1.0000
AIS	0.9248
AOS	0.6214

## Data Availability

The data are available on request from the corresponding author.

## Sample Availability

Samples are available from the authors.
